# Simvastatin and ROCK inhibition in Th0 and Th17 systems

**DOI:** 10.1186/1546-0096-10-S1-A112

**Published:** 2012-07-13

**Authors:** Josephine Isgro, Li Song, Sanjay Gupta, Alessandra B Pernis

**Affiliations:** 1 Hospital for Special Surgery, New York, NY, USA; 2 Morgan Stanley Children's Hospital of New York Presbyterian, Columbia University Medical Center, New York, NY, USA

## Purpose

The deregulation of the Def6-ROCK2-IRF4 axis in murine models results in both lupus-like and rheumatoid arthritis-like disease characterized by increased IL-17 and IL-21 production that is ameliorated by ROCK inhibition. A known beneficial pleiotropic effect of statins is inhibition of ROCK activation via their effect on RhoA activation. Statins have also been reported to decrease IL-17 and IL-21 production in T cells. Whether statins exert their inhibitory effects by interfering with the ROCK2-IRF-4 interaction in CD4+ T cells is unknown. The aim of the present study is to investigate whether statins can inhibit the ROCK pathway in CD4+ T cells and inhibit IL-17 and IL-21 production.

## Methods

Purified CD4+ T cells from the spleens and lymph nodes of wild type and Def6-deficient DO11.10 transgenic mice were stimulated with αCD3 and αCD28 in the presence/absence of simvastatin (1-10μM) with and without the known ROCK inhibitor, Y-27632 (10-30μM). Supernatants were collected and IL-17 and IL-21 production analyzed by ELISA.

## Results

As previously reported, Def6-deficient CD4+ T cells secreted significantly higher levels of IL-17 and IL-21 when stimulated as compared to wild type controls, which was ameliorated by addition of the ROCK inhibitor, Y-27632. Simvastatin significantly decreased the concentrations of IL-17 and IL-21 at all tested concentrations in a dose dependent manner (p<.05). At lower concentrations of simvastatin (<2.5μM), the addition of Y-27632 further decreased cytokine production.

## Conclusion

These data suggest that simvastatin can interfere with the ROCK pathway in CD4+ T cells and inhibit IL-17 and IL-21 production in a murine model of autoimmunity. As statins and ROCK inhibitors have distinct targets, our data furthermore suggest that combination therapy with a statin and a ROCK inhibitor may be more effective than monotherapy. Furthermore, we speculate that the decrease in cytokines is linked to a decrease in the phosphorylation status of IRF4 and its ability to target the promoters of these cytokines.

## Disclosure

Josephine Isgro: None; Li Song: None; Sanjay Gupta: None; Alessandra B. Pernis: None.

